# Disruption of axonal transport in neurodegeneration

**DOI:** 10.1172/JCI168554

**Published:** 2023-06-01

**Authors:** Sarah H. Berth, Thomas E. Lloyd

**Affiliations:** Department of Neurology, School of Medicine, Johns Hopkins University, Baltimore, Maryland, USA.

## Abstract

Neurons are markedly compartmentalized, which makes them reliant on axonal transport to maintain their health. Axonal transport is important for anterograde delivery of newly synthesized macromolecules and organelles from the cell body to the synapse and for the retrograde delivery of signaling endosomes and autophagosomes for degradation. Dysregulation of axonal transport occurs early in neurodegenerative diseases and plays a key role in axonal degeneration. Here, we provide an overview of mechanisms for regulation of axonal transport; discuss how these mechanisms are disrupted in neurodegenerative diseases including Alzheimer’s disease, Parkinson’s disease, Huntington’s disease, hereditary spastic paraplegia, amyotrophic lateral sclerosis, and Charcot-Marie-Tooth disease; and discuss therapeutic approaches targeting axonal transport.

## Introduction

Neurodegenerative diseases are a heterogeneous group of nervous system diseases, each marked by selected populations of polarized neurons undergoing synaptic retractions, axonal degeneration, and ultimately cell death. An early and unifying event in neurodegeneration is disruption of axonal transport, a constitutive process that is necessary for maintaining neuron survival.

**Figure 1:**
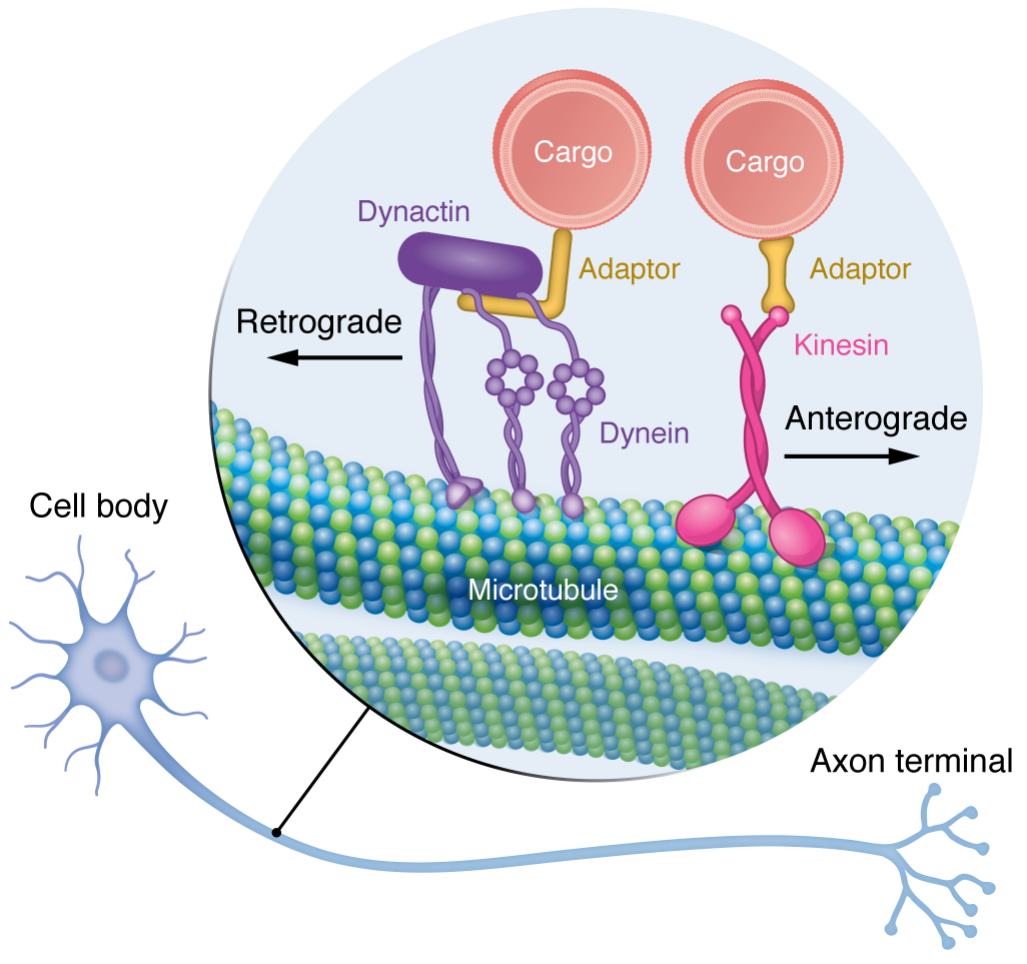
Axonal transport in neurons. Neurons are compartmentalized, with long axons. Delivery of organelles in the axon is performed via the motor proteins kinesin and dynein, which carry cargoes along microtubule tracks. Kinesin is responsible for anterograde transport of organelles, from the soma to the presynaptic terminal. Dynein is responsible for retrograde transport of organelles from the presynaptic terminal to the cell body. Dynein processivity is enhanced via binding to the essential cofactor dynactin and a cargo adaptor.

Neurons are highly polarized, with their longest axons reaching up to 1 meter in length in humans. This remarkable axonal length poses a substantial challenge to neurons to maintain homeostasis. Most synthesis occurs in the cell body, and macromolecules necessary for axonal and synaptic function are transported from the cell body and targeted to their correct destination. Conversely, signaling complexes from the synapse and macromolecules and organelles destined to be degraded or recycled are transported from the presynaptic terminal to the cell body. Neurons utilize the ATPase motor proteins kinesin and dynein, which recognize the intrinsic polarity of microtubules, to carry and deliver organelles along microtubule tracks (Figure 1).

The kinesin superfamily is composed of 44 different genes and organized into 14 different subfamilies (1). Kinesin-1 (conventional kinesin) and kinesin-3 axonal transport properties have been the best characterized (2) and will be focused on in this Review. The conventional kinesin subfamily is the most studied and consists of three proteins: *KIF5A*, which is primarily expressed in neurons, and *KIF5B* and *KIF5C*, which are expressed ubiquitously. Kinesin is composed of two heavy chains (KHCs) and two light chains (KLCs). KHC binds microtubules and hydrolyzes ATP near the N-terminus, has a long stalk region with two coiled-coiled domains, and associates with the cargo-binding KLCs at the C-terminus (1). Within axons, kinesin exclusively transports cargoes anterogradely along microtubules from the cell body to the periphery (1).

Cytoplasmic dynein, on the other hand, transports cargoes in the retrograde direction along the axon to the cell body. Cytoplasmic dynein is a large, 1.4 MDa multimeric complex composed of dimerized heavy chains (DHCs), two intermediate chains (DICs), two light intermediate chains (DLICs), and additional light chains. DHC binds microtubules and hydrolyzes ATP at its C-terminal head, and binds cargo via interaction with other dynein subunits at its N-terminal tail (1).

## Regulation of axonal transport

Axonal transport is a highly regulated process that can be modified by adaptors, by phosphorylation of motor proteins and their regulators, by posttranslational modification of microtubules, and by organelle-specific interactions. Both kinesin and dynein are autoinhibited at baseline and require activation to traffic along microtubules. Kinesin autoinhibition occurs by folding of the KHC tail to block the KHC motor from binding to microtubules (1). Dynein, on the other hand, is autoinhibited via dimerization of the motor domains, forming a structure termed the phi-particle (3). Non-dimerized dynein is in an open form that has a higher affinity for binding to microtubules but requires binding with dynactin and cargo adaptors to activate motor activity, likely by modifying the orientation of motor domains to allow processive movement along microtubules (3).

### Adaptors.

The best-characterized adaptor is dynactin, a cofactor for dynein-mediated axonal transport. Dynactin is a large, multi-subunit 1.1 MDa complex that interacts with dynein and microtubules and is essential for the initiation and activation of dynein-mediated transport (4–6). Other adaptors that function in concert with dynactin to activate dynein-mediated transport include bicaudal D proteins (7), the Hook protein family (8, 9), and spindly (10). Certain adaptors, such as TRAK2 (11), Hook3 (12), Jip1 (13), and HAP1 (14), can bind to both kinesin and dynein to regulate their trafficking (1).

### Phosphorylation.

**Figure 2:**
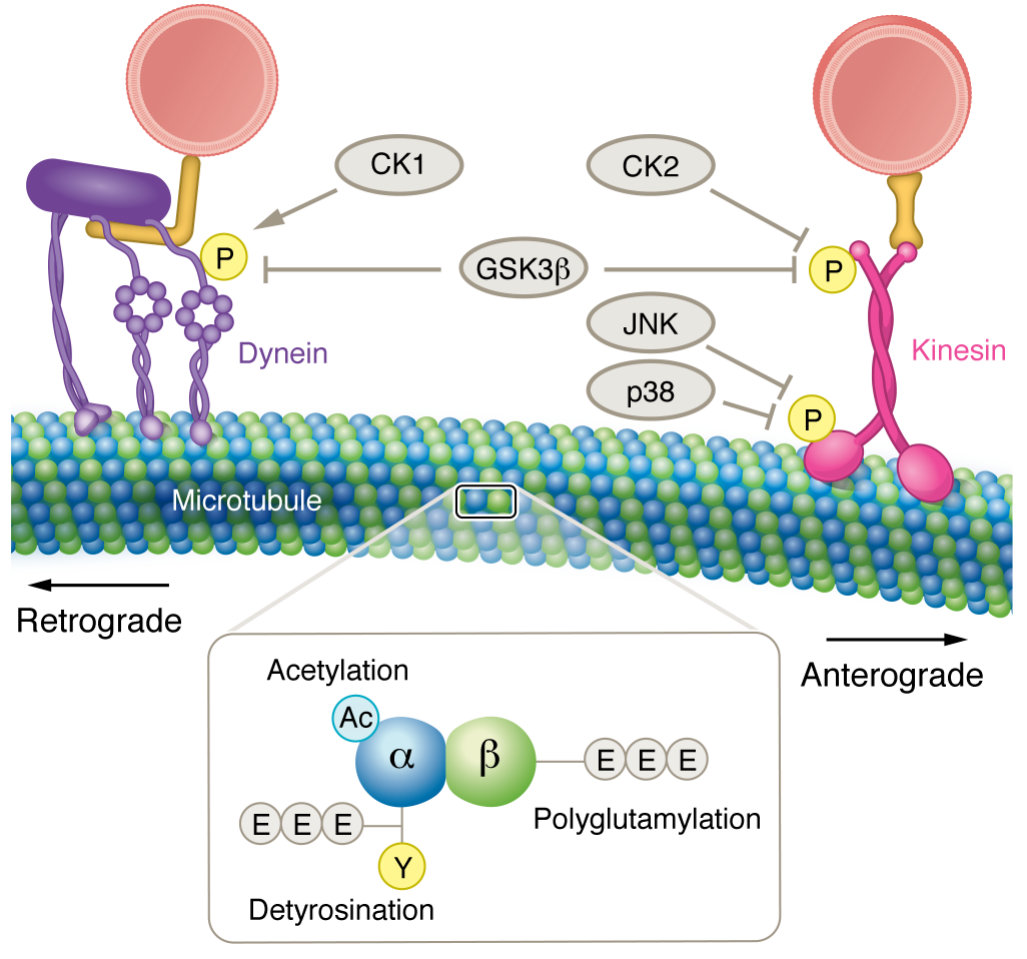
Regulation of axonal transport. Axonal transport is regulated via phosphotransferase activity. The kinases GSK3β and casein kinase 2 (CK2) inhibit anterograde axonal transport via phosphorylation of kinesin light chains, while the kinases JNK and p38 MAPK (p38) inhibit anterograde axonal transport via phosphorylation of kinesin heavy chains. GSK3β phosphorylates dynein intermediate chains to inhibit retrograde axonal transport, while CK1 phosphorylates dynein intermediate chains to activate retrograde axonal transport. Inset: Posttranslational modifications of microtubules. Microtubules are composed of α- and β-tubulin. α-Tubulin can be modified via acetylation, detyrosination, and polyglutamylation, while β-tubulin is modified by polyglutamylation.

Phosphotransferase activity regulates motor protein function (Figure 2). For example, the kinase glycogen synthase kinase-3β (GSK3β) phosphorylates KHC to inhibit axonal transport and also phosphorylates KLCs to release cargoes (15, 16). Studies using perfusion of kinases and their inhibitors into squid axoplasm showed that the stress-activated protein kinases c-Jun N-terminal kinase 3 (JNK3) and p38 mitogen-activated protein kinase (p38 MAPK) directly phosphorylate KHCs to inhibit anterograde transport (17, 18). Additionally, perfusion of active casein kinase 2 (CK2) in the squid axoplasm reduced bidirectional axonal transport velocities (18).

Dynein-mediated trafficking is also regulated by phosphorylation. Several studies have indicated that the phosphorylation of DICs can alter dynein-mediated trafficking. The kinase casein kinase 1 (CK1) phosphorylates DICs to regulate dynein-dependent transport (19). Additionally, the kinase GSK3β phosphorylates DICs to reduce DIC interaction with the adaptor NDel1, which regulates dynein motility (20). Notably, phosphorylation of DIC reduced the amount of in vitro binding of dynein to dynactin, indicating that phosphorylation may regulate interaction with this important cofactor (21).

### Microtubule regulation.

Microtubules are cytoskeletal components shaped like hollow tubes. They are composed of α- and β-tubulin, which dimerize and then polymerize into parallel protofilaments to form a microtubule. Microtubules run the entire length of the axon and form the “tracks” along which kinesin and dynein carry cargo, with the plus end oriented toward the distal axon and the minus end oriented toward the cell body. Microtubule function is highly regulated by posttranslational modifications (PTMs), such as detyrosination, polyglutamylation, and acetylation (Figure 2). This Review will focus on the latter two, as they play a role in axonal transport and have been linked to neurodegeneration.

Evidence for the role of polyglutamylation in neurodegeneration comes from *Purkinje cell degeneration* (*pcd*) mutant mice, which have neurodegeneration due to loss of function of cytosolic carboxypeptidase 1 (CCP1), a tubulin deglutaminase (22). Increasing microtubule polyglutamylation inhibits axonal transport (22–24).

Microtubule acetylation is another important regulator of axonal transport. Microtubules are acetylated by α-tubulin *N*-acetyltransferase (ATAT) and deacetylated by histone deacetylase 6 (HDAC6) and sirtuin-2 (SIRT2) (25). Acetylation weakens the lateral interactions between protofilaments, thought to confer flexibility and stabilization of microtubules (26–28). Reduction of ATAT1 led to a loss of microtubule acetylation and disruption of axonal transport (29, 30). On the other hand, increasing microtubule acetylation via increasing ATAT1 or preventing deacetylation by HDAC6 rescued axonal transport deficits in disease models (see below) (31, 32).

### Adaptors selectively regulate organelle transport.

Axonal transport regulation is tightly linked to organelle endocytosis, maturation, signaling, and degradation, indicating that axonal transport may play a role as a “hub” for integrating different cellular processes. To achieve specificity in this process, certain adaptors bind to distinct populations of cargoes, as extensively reviewed in ref. 1. For example, endolysosomal trafficking is highly regulated via specific adaptors and Rab GTPases that function as switches. For example, Rab5 controls early endosome trafficking, while Rab7 regulates late endosome maturation, motility, and fusion with lysosomes (33). Endosomal trafficking is mediated by the Hook1/dynein/dynactin complex for early endosomes, and the adaptors RILP, SKIP, and BORC for late endosomes and lysosomes (34–37).

A well-characterized population of retrogradely trafficked organelles are signaling endosomes containing neurotrophins and their receptors. Signaling endosomes are initially formed at the synapse with neurotrophin binding to receptors, and then internalized and transported to the soma for neurotrophic signaling. Signaling endosomes are specifically targeted to dynein via interactions with adaptors, including HAP1, which is important for internalization of certain neurotrophins (38); Hook1, which comigrates with signaling endosomes in the distal axon (34); and BICD1, which directs certain neurotrophins to lysosomes for degradation (39).

Lysosome transport in axons is also extensively regulated. A commonly used marker for lysosomes is LAMP1, which also labels autophagosomes and endosome pathway intermediates. A smaller percentage of LAMP1-labeled organelles in axons have acid hydrolase activity, indicating that most degradation likely occurs in or near the soma (40). Lysosomes are anterogradely transported via interactions with ARL8B and SKIP, the same adaptors that regulate anterograde transport of late endosomes, while retrograde transport is driven by the adaptors JIP3 and JIP4 (41). Autophagosome maturation and axonal trafficking are highly interlinked, as autophagosomes are generated in the distal axon and mature into autolysosomes as they are retrogradely transported (42). Autophagosome transport is regulated by the adaptors JIP1, JIP3, HTT, and HAP1 in a sequential manner as autophagosomes mature (13, 43).

Mitochondria trafficking, on the other hand, is bidirectional and is regulated via interactions between the mitochondrial outer membrane protein Miro and the motor adaptors TRAK proteins and metaxins (11, 44). Synaptic vesicle precursors are primarily trafficked anterogradely from the soma to the synapse, where they are released as mature synaptic vesicles, and are regulated via the KIF1A adaptor MADD, which activates the Rab3 GTPase on synaptic vesicles (45). Finally, dense core vesicles, which anterogradely transport neuropeptides and hormones and then undergo retrograde transport for recycling, are regulated by Arl8, Hook3, and the tyrosine phosphatase PTPN21 (45, 46).

### Axonal transport and injury signaling.

A key discovery in axonal degeneration research is the SARM1 pathway, an enzymatic pathway that uses sterile α and Toll/interleukin-1 receptor motif–containing protein 1 (SARM1) as a sensor of nicotinamide adenine dinucleotide (NAD) and its upstream activator nicotinamide mononucleotide (NMN) to drive programmed axon degeneration via NAD degradation. A key regulator of this pathway is nicotinamide mononucleotide adenylyltransferase 2 (NMNAT2), which converts NMN to NAD. NMNAT2 is synthesized in the cell body and transported into the axon. In the event of an axonal injury leading to disruption of NMNAT transport, NMN levels rise while NAD levels decrease in the axon, which activates SARM1 to induce programmed axonal degeneration (47, 48). In this way, continued anterograde axonal transport of NMNAT2 promotes axonal survival.

## Disruption of axonal transport and neurodegeneration

Although neurodegenerative diseases have diverse clinical phenotypes, a common link on a cellular level is the selective vulnerability of distinct populations of polarized neurons with elongated axons. In fact, across different neurodegenerative diseases, synaptic retractions and axonal degeneration are key early events compared with cell death (49–56). One major question regarding the role of axonal transport in neurodegeneration is whether disrupted axonal transport is an epiphenomenon of neurodegeneration, or whether dysregulation of axonal transport is a key initiator of disease. In fact, genetic evidence summarized below demonstrates that proper functioning of axonal transport is essential to maintain neuronal health.

## Mutations in motor proteins cause neurodegeneration

Underscoring the importance of fastidious regulation of axonal transport in maintaining neuronal health is that mutations in motor proteins cause neurodegeneration. For example, mutations in the KHC gene *KIF5A* cause inherited neuropathies (57), hereditary spastic paraplegia (57, 58), and amyotrophic lateral sclerosis (ALS) (59, 60). *KIF5A* mutations causing hereditary spastic paraplegia and Charcot-Marie-Tooth disease (CMT), a heterogeneous group of inherited peripheral neuropathies, are primarily missense mutations clustered in the N-terminal motor domain, whereas mutations causing ALS occur in the C-terminal cargo-binding domain, suggesting that disruption of different aspects of kinesin function underlies specificity of distinct neurodegenerative diseases (59).

Mutations in dynein have also been linked to neurodegeneration. A missense mutation in cytoplasmic dynein 1 heavy chain 1 (*DYNC1H1*) is associated with an axonal form of CMT (61). Additionally, pathogenic mutations in the *DCTN1* gene, encoding the major dynactin subunit p150^Glued^, cause neurodegeneration. A G59S mutation in *DCTN1* was first discovered to cause hereditary motor neuropathy 7B (62). Additional mutations in *DCTN1*, mostly in the CAP-Gly domain important for binding to microtubules and initiating retrograde axonal transport (5, 6), have been identified that lead to ALS (63, 64) and Perry syndrome (65), a rare disease characterized by parkinsonism, psychiatric manifestations, central hypoventilation, and weight loss.

**Figure 3:**
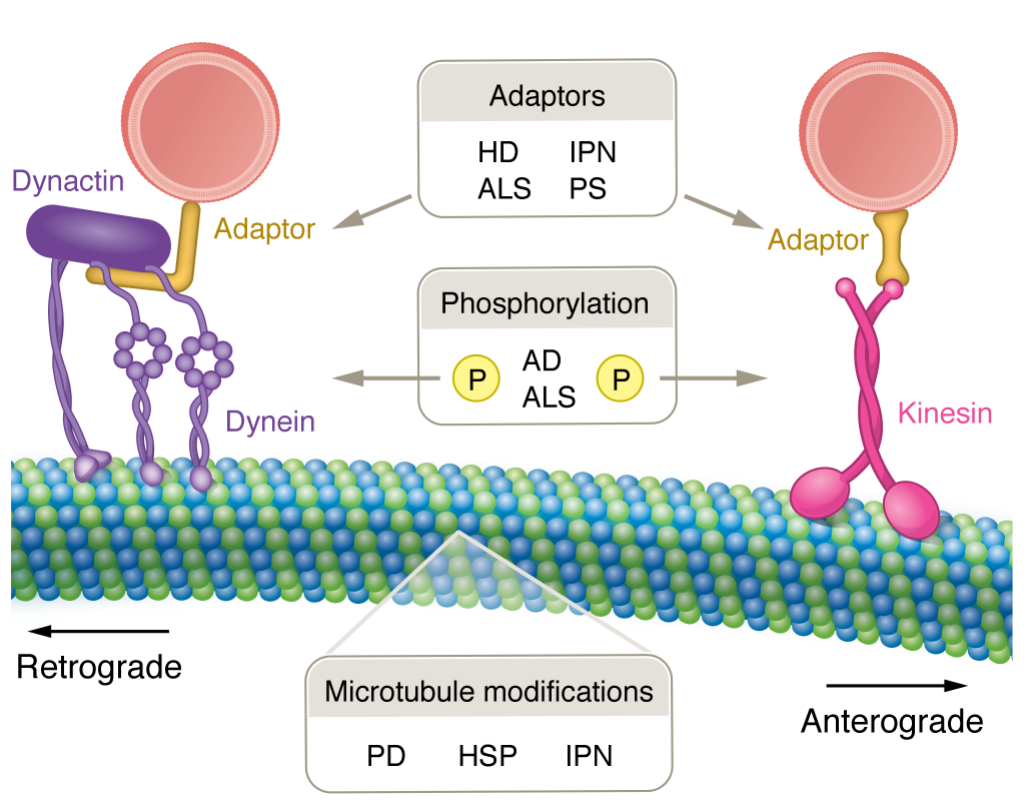
Dysregulation of axonal transport in neurodegenerative diseases. Neurodegenerative diseases disrupt axonal transport via multiple mechanisms, including motor protein regulation via phosphorylation, adaptor binding, and impaired microtubule regulation. Each neurodegenerative disease has different altered patterns of axonal transport disruption, which may contribute to disease specificity. AD, Alzheimer’s disease; ALS, amyotrophic lateral sclerosis; HD, Huntington’s disease; HSP, hereditary spastic paraplegia; IPN, inherited peripheral neuropathy; PD, Parkinson’s disease; PS, Perry syndrome.

A common link between neurodegenerative diseases is disruption of axonal transport. Below we discuss mechanisms for axonal transport disruption in specific neurodegenerative diseases, shown in Figure 3 and summarized in Table 1. Methods commonly used to assess axonal transport are detailed in Table 2.

## Alzheimer’s disease

Alzheimer’s disease (AD) is the most common cause of dementia, and pathologic hallmarks in the brain include abnormal accumulation of extracellular amyloid-β (Aβ) and intraneuronal neurofibrillary tangles (NFTs) composed of hyperphosphorylated and aggregated tau. To make Aβ, the amyloid precursor protein (APP) is cleaved first by β-secretase (BACE) and then by the protein complex γ-secretase to release Aβ fragments. Presenilin-1 (PSEN1) and PSEN2 are transmembrane proteins that compose the catalytic subunits of the γ-secretase complex. Axonal transport disruption is an early event in AD. Dystrophic axons and axonal swellings, areas of expanded axons with accumulation of cargoes and motor proteins, are found in early pathologic stages of postmortem AD brains and in an AD mouse model (49). Mouse models with familial AD mutations show axonal pathology and synaptic dysfunction before Aβ plaque formation or NFT formation (50, 51), supporting the role of early axonal dysfunction in AD. Multiple mechanisms cause impaired axonal transport in AD, including altered kinase activity and organelle-specific dysregulation.

### Altered kinase activity.

Evidence has emerged for hyperactivity of GSK3β kinase activity in AD (66), and GSK3β phosphorylates KLCs to inhibit anterograde transport (18). In AD models, perfusion of pseudophosphorylated tau into squid axoplasm to mimic hyperphosphorylated tau activated GSK3β and impaired anterograde axonal transport, implicating a role for tau phosphorylation in modulation of axonal transport (67). In fact, it was recently shown that treating primary hippocampal neurons with Aβ oligomers led to impaired KIF1A motility independent of tau, and this could be rescued by a GSK3β inhibitor (68). This indicates that GSK3β activation through multiple AD pathways can alter axonal trafficking of both kinesin-1 and kinesin-3.

### Organelle-specific dysregulation.

Postmortem AD tissue and AD mouse models have a marked increase of autophagic vacuoles and immature lysosomes within dystrophic neurites (69, 70). In addition to AD-related proteins such as APP and BACE1, axonal swellings contain marked accumulations of immature lysosomes (70). Furthermore, Aβ_1–42_ binding to DIC, resulting in disruption of the coupling between dynein and its adaptor Snapin, has been shown to impair axonal transport of amphisomes, autophagic intermediates resulting from fusion of autophagosomes with late endosomes (71). The adaptor JIP3 has also been implicated in regulating the accumulation of immature lysosomes in AD models (72). Additionally, loss of PSEN1 function impaired the acidification of amphisomes, activating JNK-mediated phosphorylation of DIC and inhibiting retrograde trafficking (73). Another proposed mechanism for lysosomal accumulation in axons in AD is impaired proteasome activity. Inhibiting proteasome activity in primary mouse neuronal cultures led to increased amyloidogenic processing of APP, thought to induce rerouting of APP into the endolysosomal pathway and disruption of lysosomal trafficking (74). Thus, different AD pathways converge on regulation of autophagolysosomal maturation and transport to specifically disrupt autophagy in axons.

## Parkinson’s disease

Parkinson’s disease (PD) is a neurodegenerative disease that prominently affects the dopaminergic cells of the substantia nigra pars compacta, leading to tremors, bradykinesia, and rigidity. α-Synuclein (αSyn), a presynaptic protein that undergoes axonal transport, is misfolded and aggregated in this disease, forming Lewy bodies. Postmortem studies and animal models have shown early axonal dysfunction, loss of presynaptic termini, and loss of motor proteins (52, 53).

There is some evidence for microtubule alterations in PD. For example, *LRRK2* mutations are the most common known genetic cause of PD and a risk factor for sporadic PD (31). In an overexpression model, mutant LRRK2 formed filamentous structures that interacted with deacetylated microtubules and disrupted axonal transport of mitochondria in rat cortical neurons and *Drosophila* (31). Restoring microtubule acetylation inhibited LRRK2 filament binding, restored axonal transport, and rescued fly locomotor deficits. However, treating primary neurons from mutant LRRK2-G2019S knockin mice with a kinase inhibitor to increase LRRK2 microtubule binding did not disrupt microtubule dynamics or axonal transport, suggesting that these filamentous structures may not alter axonal transport at endogenous levels (75).

αSyn has been shown to colocalize with microtubules both in vitro and in vivo (76, 77). In fact, αSyn promotes the formation of short microtubules, which are associated with anterograde transport of dynein. PD-associated αSyn prevented this generation of short microtubules, indicating altered direct interactions with microtubules (76). Mutant αSyn also led to a reduction of acetylated tubulin and of Kif5 within neurites (78), showing an effect on tubulin PTM as well.

### Organelle-specific dysregulation.

Loss-of-function mutations in *Parkin* and *PINK1* have been identified as causing familial PD (79, 80). PINK1 and Parkin both regulate mitophagy, the autophagic degradation of mitochondria. PINK1, a serine-threonine kinase, accumulates on damaged mitochondria, and in order to regulate mitophagy, *PINK1* mRNA is cotransported with mitochondria along axons for local translation in response to mitochondrial damage (81). Parkin is an E3 ubiquitin ligase that is recruited to mitochondria and phosphorylated by PINK1. Upon activation, Parkin ubiquitinates mitochondrial outer membrane proteins to focally remove damaged components, promote biogenesis, or, in more severe cases of mitochondrial damage, recruit adaptor proteins for mitophagy (82). In vitro studies have shown that Parkin-dependent mitophagy occurs locally in distal neuronal axons (83). Moreover, PINK1 phosphorylates Miro, a regulator of mitochondria axonal transport, to activate Parkin-dependent degradation and release mitochondria from kinesin, leading to reduced axonal transport of mitochondria (84, 85). *LRRK2* gain-of-function mutations, on the other hand, lead to an increased phosphorylation of Rab10 on depolarized mitochondria, disrupting mitophagy (86). Additionally, loss-of-function mutations of *PINK* or *Parkin*, or a gain-of-function mutation of *LRRK2*, led to resistance of the Miro1 isoform to proteasomal degradation and delayed mitophagy (87), implicating impaired mitophagy as a unifying mechanism for these PD-causing mutations. While mitophagy has been difficult to assess within in vivo mature neurons, techniques such as electron microscopy and the development of mitophagy reporter mice have aided evaluation of in vivo mitophagy in neurons (88). For example, mitophagy reporter mice expressing the LRRK-G2019S mutation have impaired basal mitophagy in dopaminergic tissues (89).

Additionally, trafficking of autophagosomes and endosomes is disrupted in PD models. For example, treatment of primary neurons with preformed αSyn fibrils caused an axonal accumulation of αSyn fibrils, which specifically impaired axonal transport of endosomes and autophagosomes (90). Further, expression of mutant LRRK2 in primary neurons reduced retrograde autophagic vesicle trafficking and impaired autophagosome maturation via recruitment of the kinesin adaptor JIP4 to increase kinesin association with autophagosomes to alter coordination of axonal trafficking (75). Intriguingly, while autophagosomes and endosomes are usually retrogradely transported in separate populations, pathogenic αSyn increased the overlap between these populations, indicating that PD may impair sorting of endosomes and autophagosomes as they undergo axonal trafficking (91).

## Huntington’s disease

Huntington’s disease (HD) is an autosomal dominant neurodegenerative disorder caused by CAG repeat expansions in the huntingtin gene (*HTT*), leading to degeneration initially in the striatum that spreads to cortical areas in the brain. Signs of HD include hyperkinetic movements such as chorea, abnormal saccades, psychiatric symptoms, and cognitive decline. HD is thought to be caused by gain-of-function and dominant-negative effects of mutant Htt, in addition to loss of wild-type Htt function (92). Axonal dysfunction and loss of axons occur early in HD before symptom onset (54, 55). Htt acts as a scaffold for axonal transport and plays critical roles in synaptic transmission and autophagy. Mutant Htt disrupts axonal transport in multiple experimental models (93–95).

Underscoring the importance of adaptors for proper regulation for axonal transport is the finding that Htt itself acts as an adaptor for axonal transport by binding to DIC to facilitate dynein-mediated transport (96). In fact, Htt has been implicated in axonal transport of a broad range of vesicles (43, 97–105) and has been suggested to promote axonal transport (94, 103, 106). Htt interacts with another adaptor, huntingtin-associated protein 1 (HAP1), which interacts with the p150^Glued^ subunit of dynactin, DLIC, KIF5C, and KLC to regulate axonal transport (1, 14). Mutant Htt disrupts function of the Htt/HAP1 complex (106) to impair axonal transport (94, 103, 106).

Additionally, Htt is regulated via PTMs, and altered PTM regulation of mutant Htt may play an important role in HD pathogenesis. Htt is phosphorylated by the kinase Akt at S421 to promote anterograde trafficking of APP, and this Akt/Htt pathway has been shown to be downregulated in HD brains (107). Furthermore, Htt is dimethylated by protein arginine methyltransferase 6 (PRMT6) to facilitate axonal transport, and *S*-adenosylhomocysteine, which regulates PRMT6, is downregulated in HD models. Overexpressing PRMT6 in mutant Htt cell culture and fly models was able to rescue phenotypes, indicating that decreased dimethylation of mutant Htt may alter Htt’s regulation of axonal transport in HD (108).

## Hereditary spastic paraplegia

Hereditary spastic paraplegia (HSP) is a group of inherited diseases with progressive spasticity in the legs due to axonal degeneration of upper motor neurons and dorsal columns (109). Mutations in over 73 genes have been described that cause HSP (110). These genes have been implicated in multiple pathways, including axonal transport, metabolic pathways, ER dynamics, vesicle formation, myelination, and autophagy (110). In fact, close to 10% of patients with HSP have *KIF5A* mutations, most of which are in the motor domains, leading to reduced kinesin motility (58, 111, 112). Other HSP-causing mutations, including mutations in *SPAST* (which encodes spastin), *SPG11* (encoding spatacsin), and *ZYFE26* (encoding spastizin), also impair axonal transport, showing that this is likely a common mechanism for HSP (113–116).

The most common autosomal dominant cause of HSP is mutations in *SPAST*, encoding spastin, an AAA ATPase that severs polyglutamylated microtubules, forming shorter microtubule fragments (117). Depletion of *SPAST* led to longer microtubules, increased polyglutamylation, and reduction of kinesin-mediated axonal transport (118). *SPAST* mutations also reduced tubulin acetylation in patient-derived stem cell models (119). In fact, mutant spastin models showed impaired axonal transport of multiple organelles (113, 120–122), indicating that spastin-mediated severing of microtubules likely affects axonal transport function. Further, loss of spastin caused reduced microtubule dynamics and density, underscoring its importance in axonal transport (123–125).

## Amyotrophic lateral sclerosis

Amyotrophic lateral sclerosis (ALS) is a neurodegenerative disease of upper and lower motor neurons, leading to progressive weakness, spasticity, and hyperreflexia. Early pathologic studies of postmortem tissue showed axonal swellings and neurofilament accumulation, consistent with axonal transport defects (56). While close to 90% of ALS is sporadic, mutations in over 25 genes cause familial ALS. Underscoring impaired axonal transport as a pathogenic mechanism is that mutations in genes encoding multiple motor proteins, including the kinesin gene *KIF5A*, the dynactin gene *DCTN1*, and the dynein gene *DYNC1H1*, cause ALS (59, 63, 64, 126, 127). Axonal transport defects have been further identified across multiple models of genetic forms of ALS (128). For example, axonal transport is perturbed by mutations in *SOD1* (encoding superoxide dismutase 1) that cause ALS (129–131). Likewise, ALS-causing mutations of FUS and TDP-43 also alter axonal transport (132–134). Potential mechanisms for these impairments are discussed below.

### Altered kinase activity regulating axonal transport.

Mutant FUS, mutant SOD1, and oxidized SOD1 inhibited axonal transport in squid axoplasm via activation of the stress-activated protein kinase p38 MAPK, which phosphorylated KHCs to release kinesin from microtubules (135–137). Impairment of axonal transport was rescued by inhibition of p38 MAPK in squid axoplasm, primary neurons, and mouse models of SOD1-mutant ALS (135, 136, 138).

A CRISPR/Cas9 screen identified NEK6, a kinase known to regulate the microtubule-based mitotic spindle, as a mediator of poly-proline-arginine toxicity (139). Depletion of NEK6 was sufficient to rescue axonal transport deficits in cortical neurons derived from C9orf72 patient induced pluripotent stem cells (139). Knocking down NEK6 altered phosphorylation of multiple proteins associated with axonal transport, indicating that NEK6 activity modulates axonal transport via phosphorylation of axonal transport regulators (139).

### Organelle-specific dysregulation.

ALS mutations have varied effects on vesicle trafficking. First, mitochondria transport was impaired in mutant SOD1 models of ALS (140). SOD1 mutations led to a reduction of Miro levels; overexpression of Miro1 was able to rescue impaired axonal transport in mutant SOD1 cortical and motor neurons (141). Disrupted mitochondrial trafficking as a pathogenic mechanism has also been proposed for ALS caused by a GGGGCC hexanucleotide repeat expansion (HRE) in the *C9orf72* gene, which is the most common genetic cause of ALS. A pathogenic mechanism for *C9orf72* ALS is via repeat-associated non-AUG translation of the HRE transcripts, creating aggregation-prone and neurotoxic dipeptide repeat (DPR) proteins, including poly-glycine-arginine (poly-GR) and poly-proline-arginine (poly-PR). Although mitochondrial trafficking was not altered in motor nerves in a third-instar larva *Drosophila* model of *C9orf72* ALS (142), another recent study found reduced mitochondrial trafficking in aged, induced pluripotent stem cell–derived (iPS-derived) motor neurons and adult *Drosophila* neurons (143). Single-molecule tracking showed that poly-GR and poly-PR DPRs associated with the C-terminal tubulin tails of microtubules and caused motor pausing or detachment to reduce axonal transport (143). It is plausible that disrupted mitochondrial trafficking may be an age-related phenomenon in *C9orf72* ALS.

Intriguingly, even at a young age, in vivo motor neurons in *Drosophila* had markedly impaired autophagosome biogenesis, late endosome trafficking, and an accumulation of static lysosomes, indicating that early disruption in the axonal autophagolysosomal pathway may be a key pathogenic event in *C9orf72* ALS (142, 144). While there is more evidence for a toxic gain of function by *C9orf72* HRE, loss of function of *C9orf72* due to the HRE may also contribute to pathogenesis. Indeed, C9orf72 localizes with Rab5-positive early endosomes, and loss of function of C9orf72 disrupted early endosomal maturation and trafficking in iPS motor neurons (145). Other evidence for certain ALS mutations having specific effects on axonal transport is in vivo analysis showing impaired axonal transport of signaling endosomes specifically in mutant TDP-43 mice but not mutant FUS mice (146). Finally, there has been growing interest in how axonal transport of mRNA, which is important for local translation in axons, is impaired in ALS. In fact, TDP-43 is cotransported with messenger ribonucleoprotein granules (comprising nontranslating mRNA and bound proteins), indicating that TDP-43 may regulate this process (147). Disruption of mRNA axonal transport has been associated with annexin A11 (ANXA11) mutations causing ALS (148). ANXA11 tethers RNA granules to trafficking lysosomes; ALS-causing ANXA11 mutations disrupted these interactions to reduce ANXA11 binding to RNA granules and to lysosomes (149).

## Inherited peripheral neuropathies

Inherited peripheral neuropathies are characterized by progressive degeneration of the peripheral nerves. CMT, one of the most common inherited neurologic diseases, is a heterogeneous group of peripheral neuropathies with progressive distal weakness, numbness, and atrophy. Over 100 causative genetic mutations have been discovered thus far (150), including mutations in *DCTN1*, implicating impaired axonal transport as an underlying mechanism (62). Other genetic mutations impair axonal transport via alterations in microtubules and their PTMs, as well as via disruption of axonal trafficking of specific organelles.

Mutations in the *TUBB3* gene, which encodes β-tubulin isotype III, cause a hereditary axonal polyneuropathy; mouse models of disease-causing *TUBB3* mutations had impaired microtubule dynamics with an increase of microtubule polyglutamylation and acetylation (151). Additionally, certain mutations in *HSPB1* cause CMT2F and distal hereditary motor neuropathy (152). These mutations had an increased binding to tubulin and microtubules, leading to altered microtubule dynamics via stabilization of microtubules (153). *HSPB1* also impairs microtubule acetylation and axonal transport; inhibiting HDAC6 to increase microtubule acetylation improved axonal transport deficits (154, 155). Further, a CMT-linked dominant mutation of *GARS* led to aberrant interactions between glycyl-tRNA synthetase and HDAC6, leading to a decrease in microtubule acetylation; treating with an HDAC6 inhibitor restored this deficit (156).

### Organelle-specific dysregulation.

Several CMT-causing mutations impair axonal transport of mitochondria. Mitofusin 2 (MFN2) is a mitochondrial outer membrane protein important for mitochondrial fusion, and CMT-causing mutations in *MFN2*, which encodes MFN2, are the most common cause of axonal CMT (157). These mutations induce impaired axonal transport of mitochondria, likely through direct interactions with Miro, a mitochondrial protein that regulates attachment of mitochondria to the motor proteins in a calcium-dependent manner (158–160). Additionally, CMT-causing mutations in the cation channel transient receptor potential vanilloid 4 (*TRPV4*) led to an increase in intracellular calcium and impaired mitochondrial trafficking via dysregulation of Miro (161, 162). Further, expression of mutant *HSPB1* in primary motor neurons induced mitochondrial abnormalities and disrupted axonal trafficking of mitochondria to a greater extent than other organelles (163). Another CMT-linked gene that has been related to impaired mitochondrial trafficking is *GAN*, encoding gigaxonin, an E3 ligase adaptor that regulates intermediate filaments. Intermediate filament aggregation caused by mutant *GAN* led to impaired mitochondrial motility, possibly due to impaired intermediate filament regulation of mitochondrial distribution (164, 165). Additionally, CMT-causing mutations in the *NEFL* gene (encoding neurofilament light chain) led to impaired trafficking of both neurofilament light chains and mitochondria (159, 166–168). Finally, again showing the important link between endosomal maturation and axonal transport are CMT-causing mutations in *RAB7*, which encodes the Rab7 GTPase on late endosomes that regulates trafficking. These CMT-causing Rab7 mutations caused impaired axonal transport of endosomes; biochemical assays revealed that these disease-causing mutations led to excess activation and misregulation of Rab7 activity (169–171).

## Axonal transport–directed therapeutics

Attention has been turned to targeting aberrant kinase activity to treat neurodegeneration. Of the kinase inhibitors shown to modify axonal transport discussed above, clinical trials to evaluate GSK3β and p38 MAPK in neurodegeneration have been initiated. Two GSK3β inhibitors have been used in clinical trials for AD. A pilot study of Tideglusib treatment in AD showed a positive trend in cognition without significance (172), but a phase II clinical trial in AD did not show clinical benefit (173). A phase I clinical trial of the GSK3β inhibitor AZD1080 showed target engagement, but no further clinical trials evaluating AZD1080 in neurodegenerative disease have been published (174). p38 MAPK inhibitors have also been targeted as treatment for neurodegenerative disease. A phase IIa study of the p38 MAPKα inhibitor neflamapimod in patients with early AD showed a significant improvement in episodic memory (175). However, a subsequent phase II trial with 24-week treatment with neflamapimod (REVERSE-SD) did not replicate these results (176).

HDAC6 inhibitors have also been considered as a therapeutic target to increase microtubule acetylation and promote axonal transport. Thus far, there have been conflicting reports regarding the extent of microtubule acetylation defects in neurodegeneration. For example, in AD, there have been reports of either increased or decreased α-tubulin acetylation (177, 178). Additionally, while it has been shown that tubulin acetylation is decreased in HD, correcting this defect in a mouse model did not alter disease progression (179). However, HDAC6 inhibitors to increase microtubule acetylation have been shown to rescue phenotypes across models of multiple neurodegenerative diseases with impaired axonal transport, such as AD, PD, HD, ALS, and CMT (31, 132, 154, 156, 180–188). Note that besides α-tubulin, HDAC6 has other cytosolic targets, including tau, heat shock protein 90, cortactin, peroxiredoxin, and heat shock transcription factor-1, and the development of inhibitors to solely affect microtubule acetylation in the future will allow for greater specificity (180, 189). The HDAC6 inhibitor vorinostat is currently in a phase Ib study for patients with AD (ClinicalTrials.gov NCT03056495), and the HDAC6 inhibitor nicotinamide is currently in a phase II trial for AD (NCT03061474).

## Conclusions

While axonal transport is clearly disrupted in neurodegeneration, major questions still remain. One is whether disruption of axonal transport is an epiphenomenon in the setting of diseased neurons, as opposed to a key pathogenic event. There is stronger evidence for disrupted axonal transport as a key pathogenic event in peripheral neuropathies and motor neuron diseases, as genetic mutations in motor proteins themselves cause these diseases. It is difficult to exclude the possibility that these mutations may exert deleterious effects outside of axons, for example on organelle trafficking in the soma or dendrites. However, peripheral nervous system neurons have the longest axons in the body, and are thus particularly reliant on axonal transport for homeostasis. Supporting this hypothesis is that disruption of axonal trafficking via certain chemotherapeutic drugs such as paclitaxel causes peripheral neuropathies, demonstrating the importance of proper functioning of axonal transport in preventing neurodegeneration.

Axonal transport regulation is complex and regulated through multiple synergistic mechanisms, and alterations in axonal transport in specific diseases are likely due to different patterns of dysregulation of specific components including motor proteins, microtubules, and cargoes. As axonal transport declines with age, it is plausible that initially neurons are able to compensate for defects in axonal transport. However, as axonal transport declines and more targets are dysregulated, neurons become unable to compensate for these defects, leading to axonal degeneration. Thus, targeting therapies at early stages of axonal transport disruption would be important to prevent the subsequent pathogenic cascade in neurodegeneration.

Additionally, while axonal transport has clearly been shown to be disrupted in neurodegeneration, other cellular functions such as autophagy and nucleocytoplasmic transport are also impaired in neurodegeneration. Evidence continues to emerge that these distinct cellular processes are extensively interconnected. In fact, axonal transport likely acts as a signaling hub to coordinate organelle maturation and trafficking to affect diverse cellular processes, giving credence to the importance of axonal transport to maintain homeostasis in the axon. It is plausible that treatment of neurodegeneration will require a multimodal approach, targeting different aspects of cellular function to protect neurons. Directing therapeutics at axonal transport may be crucial to stabilize axonal function in conjunction with other disease-modifying therapies. Thus far, clinical trials using kinase inhibitors and HDAC6 inhibitors to treat neurodegeneration have begun, although their treatment efficacy remains to be determined. Continued, detailed assessments of precise mechanisms underlying axonal transport deficits will be beneficial for further development of therapeutic targets.

## Figures and Tables

**Table 1:**
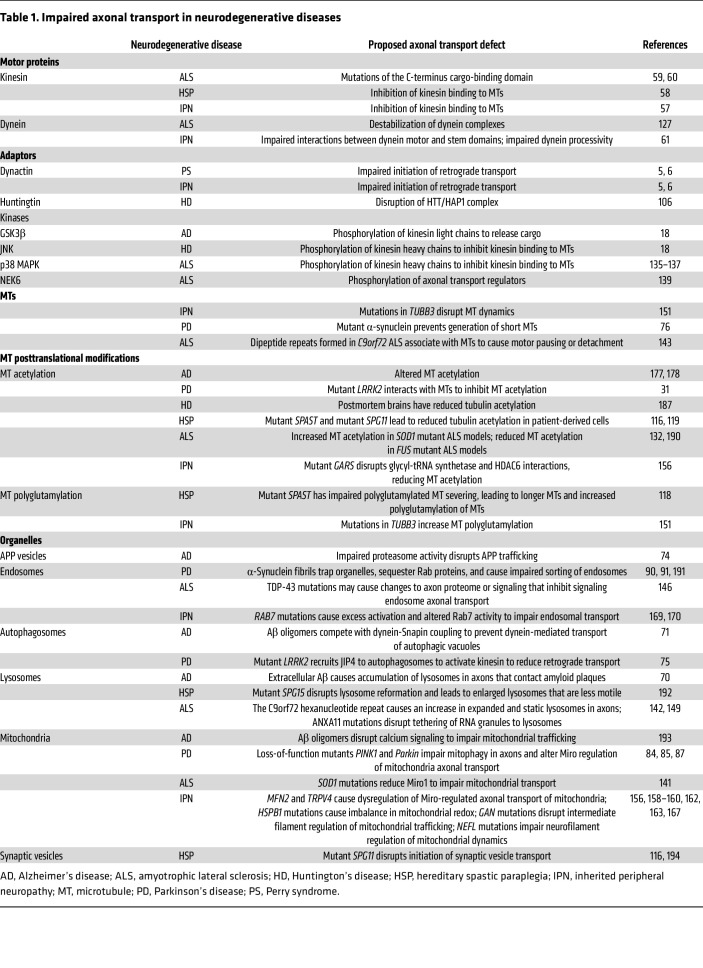
Impaired axonal transport in neurodegenerative diseases

**Table 2:**
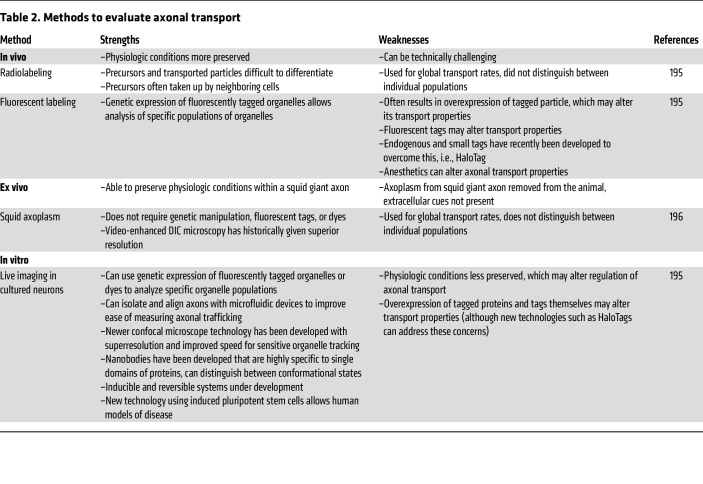
Methods to evaluate axonal transport

## Version 1 06/01/2023

Electronic publication
